# Association of high cost sharing and targeted therapy initiation among elderly Medicare patients with metastatic renal cell carcinoma

**DOI:** 10.1002/cam4.1262

**Published:** 2017-12-01

**Authors:** Pengxiang Li, Yu‐Ning Wong, Jordan Jahnke, Amy R. Pettit, Jalpa A. Doshi

**Affiliations:** ^1^ Division of General Internal Medicine Department of Medicine Perelman School of Medicine University of Pennsylvania Philadelphia Pennsylvania; ^2^ Leonard Davis Institute of Health Economics Philadelphia Pennsylvania; ^3^ Fox Chase Cancer Center Philadelphia Pennsylvania; ^4^ Center for Public Health Initiatives University of Pennsylvania Philadelphia Pennsylvania

**Keywords:** Cost sharing, Medicare, renal cell carcinoma, specialty drugs, targeted therapies

## Abstract

High out‐of‐pocket costs may limit access to oral therapies covered by patients’ prescription drug benefits. We explored financial barriers to treatment initiation in patients newly diagnosed with metastatic renal cell carcinoma (mRCC) by comparing Medicare Part D patients with low out‐of‐pocket costs due to receipt of full low‐income subsidies (LIS beneficiaries) to their counterparts who were responsible for more than 25% cost sharing during Medicare's initial coverage phase (non‐LIS beneficiaries). We used 2011–2013 100% Medicare claims for non‐LIS and LIS beneficiaries newly diagnosed with metastases in the liver, lung, or bone to examine targeted therapy treatment initiation rates and time to initiation for (1) oral medications (sorafenib, sunitinib, everolimus, pazopanib, or axitinib) covered under Medicare's prescription drug benefit (Part D); (2) injected or infused medications (temsirolimus or bevacizumab) covered by Medicare's medical benefit (Part B); and (3) any (Part D or Part B) targeted therapy. The final sample included 1721 patients. On average, non‐LIS patients were responsible for out‐of‐pocket costs of ≥$2,800 for their initial oral prescription, as compared to ≤$6.60 for LIS patients. Compared to LIS patients, a lower percentage of non‐LIS patients initiated oral therapies (risk‐adjusted rates, 20.7% vs. 33.9%; odds ratio [OR] = 0.49, 95% CI: 0.36–0.67, *P *<* *0.001) and any targeted therapies (26.7% vs. 40.4%, OR = 0.52, 95% CI: 0.38–0.71, *P *<* *0.001). Non‐LIS patients were also slower to access therapy. High cost sharing was associated with reduced and/or delayed access to targeted therapies under Medicare Part D, suggesting that financial barriers play a role in treatment decisions.

## Introduction

Research advances have led to increased therapeutic options for many oncology patients, yet these treatments are only beneficial if individuals can access them. While clinic‐ or hospital‐based intravenous chemotherapy is typically covered by a patient's medical benefit, many newer drugs are self‐administered oral agents that are covered under the prescription benefit. Given the expense of many newer treatments, they are frequently associated with cost sharing that leads to considerable out‐of‐pocket costs for patients 1—raising important questions about whether cost sharing represents a barrier to treatment initiation.

This is especially relevant for the treatment of metastatic renal cell carcinoma (mRCC). A rapid pace of drug development significantly altered the treatment paradigm for mRCC, and there are now 10 FDA‐approved targeted agents, seven of which are oral medications (Table 1). These expanded treatment options have demonstrated reduced toxicity and been shown to extend survival in clinical trials 2, 3, 4, more than doubling the median overall survival of approximately 1 year conferred by pretargeted era therapies 2, 5, 6, 7, 8, 9.

**Table 1:** FDA‐approved targeted therapies for the treatment of advanced renal cell carcinoma^a^

Generic name	Brand name	Route of administration	Covered under Medicare Part D or B	FDA approval date	Included in the study
Sorafenib	Nexavar	Oral	D	Dec 2005	Yes
Sunitinib	Sutent	Oral	D	Jan 2006	Yes
Temsirolimus	Torisel	Intravenous	B	May 2007	Yes
Everolimus	Afinitor	Oral	D	Mar 2009	Yes
Bevacizumab	Avastin	Intravenous	B	July 2009	Yes
Pazopanib	Votrient	Oral	D	Oct 2009	Yes
Axitinib	Inlyta	Oral	D	Jan 2012	Yes
Nivolumab	Opdivo	Intravenous	B	Nov 2015	No
Cabozantinib	Cabometyx	Oral	B	Apr 2016	No
Lenvatinib	Lenvima	Oral	D	May 2016	No

a All FDA‐approved drugs that were available during the study period (2011‐2013) were included.

Cost‐sharing requirements for the oral targeted treatments are complex and high, particularly for patients covered by Medicare Part D who are not eligible for low‐income subsidies (non‐LIS beneficiaries). Per Center for Medicare and Medicaid Services regulations, Part D prescription drug plans may place any drug that exceeds a designated cost threshold ($600/month from 2011 to 2015) on a “specialty tier,” which typically requires patients to pay 25–33% coinsurance during each calendar year's initial coverage phase 10. Once patients’ total drug spending exceeds an initial coverage limit ($2840–$2960 from 2011 to 2015) 11, they enter a coverage gap phase requiring even higher cost sharing (45–50% coinsurance from 2011 to 2015). After their total out‐of‐pocket Part D spending reaches a limit ($4550–$4700 from 2011 to 2015) that triggers catastrophic coverage, patients pay 5% coinsurance for the remainder of that calendar year 11. In contrast, patients who meet Medicare eligibility requirements for receipt of full low‐income subsidies (LIS) are responsible for cost sharing of ≤$6.60 per month.

We examined the association of high out‐of‐pocket costs under Medicare Part D with targeted therapy initiation in the 6 months following mRCC diagnosis. Utilizing LIS beneficiaries as a natural control group, we examined this issue in three ways. First, we determined whether non‐LIS patients had lower initiation rates for Part D targeted therapies compared to LIS patients, who faced nominal copayments. Second, since infusible targeted therapies are covered by Medicare's medical benefit (Part B) and thereby associated with relatively modest out‐of‐pocket costs for most non‐LIS patients 12, we examined whether non‐LIS patients showed higher initiation rates for those therapies, when compared to LIS patients who faced similar costs for both Part D and Part B medications. Third, we assessed the overall association between LIS status and any (Part D or B) targeted therapy initiation.

## Methods

### Study design

This retrospective claims‐based study examined targeted therapy initiation among elderly fee‐for‐service Medicare beneficiaries. We compared initiation among beneficiaries subject to high levels of cost sharing under Part D at the time of initial mRCC diagnosis (non‐LIS group) to a contemporaneous comparison group of newly diagnosed patients with full LIS, who faced nominal cost sharing for the same medications (LIS group). Patients receiving partial LIS were not included.

### Data source

We used a data extract of the 2011–2013 100% Chronic Conditions Data Warehouse (CCW) Medicare claims, which contain data on all fee‐for‐service Medicare beneficiaries in the U.S., linked with Part D plan and formulary characteristics files for patients with ≥1 diagnosis of RCC (ICD‐9‐CM code 189.0) during these years.

### Sample selection

We applied additional inclusion criteria to identify patients who were newly diagnosed with mRCC: (1) ≥1 inpatient or outpatient claim indicating metastatic disease (ICD‐9‐CM codes 196–199) between July 1, 2011 and June 30, 2013, the first of which represented the “index date”; (2) first metastatic site in the liver (197.7), lung (197.0), or bone/bone marrow (198.5); (3) continuous enrollment in both fee‐for‐service Medicare and a stand‐alone Part D prescription drug plan for 180 days before and after the index date (pre‐index period and post‐index period, respectively); (4) ≥2 claims with a diagnosis of metastatic disease, occurring ≥30 days apart (i.e., the index claim and at least one other claim during the post‐index period), to decrease the likelihood of including patients who later received a revised diagnosis; (5) ≥2 claims for RCC occurring ≥30 days apart, as further confirmation of diagnostic status; (6) index date during the beneficiary's Part D initial coverage phase; and (7) age ≥65 years on the index date.

**Figure A1:**
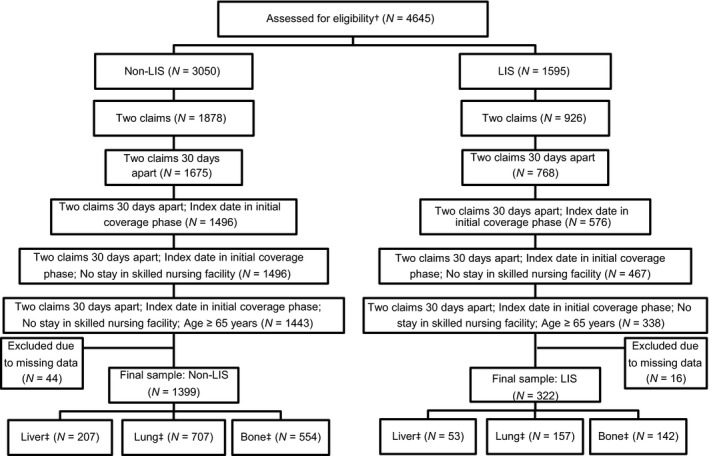
Sample selection flow chart. ^†^Initial eligibility criteria were: Medicare beneficiaries with ICD‐9 codes for renal cell carcinoma and first metastasis in liver, lung, or bone; with fee‐for‐service Part D plan coverage 6 months before and after the date of the first metastasis claim (index date); without missing data for LIS status; and without a targeted therapy claim 6 months before the index date. ^‡^Refers to site of initial metastasis. Categories are not mutually exclusive; patients were assigned to multiple categories if the first date of reported metastatic diagnosis in medical claims included multiple sites.

Patients were excluded if they had: (1) any metastatic claim during the pre‐index period; (2) any claim for a targeted therapy during the pre‐index period; (3) any change in LIS status; (4) a stay in a skilled nursing facility during the pre‐ or post‐index period (our data do not capture prescription drug use during these stays); or (5) missing data for important covariates. Figure A1 shows a sample selection diagram.

Selection criteria were designed to capture mRCC patients likely to be appropriate candidates for targeted therapy. We focused on patients with initial metastases in three of the most common metastatic sites for RCC 13 and excluded sites where patients would be more likely to have indolent disease that may warrant observation as an initial treatment approach (lymph node only) or where patients are often treated with upfront radiation therapy and/or surgery rather than systemic treatment (brain only) 14, 15, 16.

Additionally, our sample focused on patients who were newly diagnosed during Part D's initial coverage phase, for two reasons. First, focusing on a single Part D coverage phase meant patients would all be facing the same cost‐sharing level at the time of mRCC diagnosis. Second, since patients who had already moved out of this coverage phase would have done so because of spending on other multiple and/or expensive medications, we improved our ability to isolate the impact of financial burden related to the mRCC specialty drug specifically. Less than 15% of potentially eligible patients were diagnosed outside of the initial coverage phase.

### Outcome variables

Our main outcome variable was defined as the percentage of patients with a claim for a targeted therapy within 6 months of mRCC diagnosis (index date). We included all FDA‐approved targeted therapies available during the study period (Table 1). We separately examined fills for any Part D targeted therapies, administrations for any Part B targeted therapies, and either. Targeted therapies were identified from Part D prescription claims via National Drug Codes and Part B medical claims using Healthcare Common Procedure Coding System codes.

In addition, we examined time to initiation for Part D, Part B, and any targeted therapy, defined as the number of days elapsed between the index date and the date that the first Part D, Part B, or any targeted therapy was filled or administered during the 6‐month post‐index period. Patients who did not have a targeted therapy claim during the post‐index period were considered censored.

### Statistical analyses

Descriptive statistics were generated for the main sample. Multivariable logistic regressions were used to examine differences in targeted therapy initiation between non‐LIS and LIS patients. Model covariates included sociodemographic characteristics capturing age, sex, race, and region; clinical characteristics including site of first metastasis, whether there were multiple sites when the first metastatic claim was identified, and Charlson Comorbidity Index score 17; and plan characteristics including Part D drug benefit type, targeted therapy formulary coverage, and utilization management tools. Zip code‐level median household income and percentage of individuals aged 25 or older with at least a high school degree were included as proxies for socioeconomic status. Finally, we included indicators for the index date year to control for any temporal trends. Huber–White (robust) standard errors were used to adjust for plan formulary‐level clustering.

In addition, we performed sensitivity analyses to test the robustness of our results. First, we used Part D plan formulary fixed‐effects conditional logistic regressions. This allowed us to compare non‐LIS and LIS patients from within the same plan formulary, thereby ruling out the influence of other formulary‐related restrictions (e.g., prior authorization), so as to isolate the effects of cost‐sharing differences. Second, we included all patients meeting our main sample selection criteria, even if they were diagnosed after the initial coverage phase. Third, we relaxed our criteria for identifying new mRCC patients by only requiring ≥2 metastatic claims, regardless of whether they occurred ≥30 days apart. Fourth, we included patients with a stay in a skilled nursing facility. Fifth, we repeated our analysis controlling only for statistically significant covariates. In addition, we used Kaplan–Meier curves and multivariable Cox regressions (adjusting for covariates listed above) to examine the difference in time to targeted therapy initiation between non‐LIS and LIS patients.

All statistical analyses were performed using SAS 9.4 and STATA/MP 14. The University of Pennsylvania Institutional Review Board deemed the study exempt from informed consent procedures.

## Results

Baseline characteristics are presented in Table 2. Our selection criteria identified 1721 patients. Although the two groups were similar overall, the non‐LIS group had a higher percentage of males and white patients and a lower mean Charlson Comorbidity Index score. In addition, the non‐LIS group was more likely to be in a plan that covered a higher proportion of the available targeted therapies and more likely to live in zip codes with higher median household income and with a greater percentage of individuals with at least a high school degree.

**Table 2:** Sample characteristics

Characteristic	Non‐LIS	LIS	*P*‐value^a^
	(N = 1399)	(N = 322)	
Mean age (SD), years	75.2 (6.4)	74.6 (6.7)	0.170
Sex, No. (%)			
Female	536 (38.3%)	147 (45.7%)	0.016
Male	863 (61.7%)	175 (54.3%)	
Race/Ethnicity, No. (%)			
White	1261 (94.5%)	189 (64.3%)	<0.001
Black	50 (3.7%)	47 (16.0%)	
Hispanic and Other^b^	23 (1.7%)	58 (19.7%)	
Region, No. (%)			
North	244 (17.4%)	51 (15.8%)	<0.001
Midwest	394 (28.2%)	58 (18.0%)	
South	549 (39.2%)	147 (45.7%)	
West	212 (15.2%)	66 (20.5%)	
Site of first metastasis, No. (%)^c^			
Liver	207 (14.8%)	53 (16.5%)	0.440
Lung	707 (50.5%)	157 (48.8%)	0.560
Bone	554 (39.6%)	142 (44.1%)	0.140
First metastases involved multiple sites, No. (%)	248 (17.7%)	67 (20.8%)	0.200
Mean Charlson Comorbidity Index score (SD)	0.82 (1.28)	1.01 (1.36)	0.019
Part D drug benefit type, %^d^			
Basic alternative	183 (13.1%)	203 (63.0%)	<0.001
Enhanced alternative	440 (31.5%)	11 (3.4%)	
Defined standard benefit and Other	776 (55.5%)	108 (33.5%)	
Part D plan formulary characteristics			
Proportion (SD) of targeted therapies on market covered on the plan formulary	0.97 (0.06)	0.92 (0.08)	<0.001
Proportion (SD) of covered targeted therapies requiring prior authorization	0.88 (0.18)	0.86 (0.27)	0.140
Proportion (SD) of covered targeted therapies subject to quantity limits	0.35 (0.39)	0.33 (0.35)	0.590
Proportion (SD) of covered targeted therapies subject to step therapy	0.00 (0.00)	0.00 (0.00)	
Zip code‐level variables			
Median (SD) household income, $10,000s	5.82 (2.23)	4.74 (1.73)	<0.001
Percentage (SD) of those ≥25 years with at least a high school degree	87.41 (8.23)	79.72 (11.93)	<0.001
Year of first mRCC diagnosis, No. (%)			
2011	403 (28.8%)	88 (27.3%)	0.520
2012	685 (49.0%)	153 (47.5%)	
2013	311 (22.2%)	81 (25.2%)	

LIS, low‐income subsidy; mRCC, metastatic renal cell carcinoma; SD, standard deviation.

a Statistical comparisons used ANOVA for continuous variables and Pearson's chi‐square tests for categorical variables.

b Per CMS data use agreement specifications, these groups were combined due to small cell size in the Hispanic category.

c Categories are not mutually exclusive; patients were assigned to multiple categories if the first date of reported metastatic diagnosis in medical claims included multiple sites.

d Defined standard benefit has an annual deductible, 25% coinsurance in the initial coverage phase, and 45% cost sharing during the coverage gap; basic alternative may have reduced or $0 deductible, can use tiered copayments or coinsurance, and must be actuarially equivalent to the defined standard benefit; enhanced alternative exceeds the value of standard coverage and may include reduction/elimination of the initial deductible, an increase in the initial coverage limit, or a reduction of cost sharing in the coverage gap.

Given mean total costs of ~$7200 per 30‐day prescription for targeted therapies covered by Part D, non‐LIS patients’ first 30‐day fill “straddled” Part D benefit phases (pushing beneficiaries out of the initial coverage phase and into the coverage gap phase) and generated out‐of‐pocket costs of ≥$2800 (data not shown). On the other hand, LIS patients faced out‐of‐pocket costs of ≤$6.60 (data not shown).

As shown in Table 3, a lower percentage of the non‐LIS group initiated Part D therapies, as compared to the LIS group (risk‐adjusted rates 20.7% vs. 33.9%; odds ratio [OR] = 0.49, 95% CI: 0.36–0.67, *P *<* *0.001). Initiation rates for Part B therapies were similar across groups (8.2% vs. 10.2%, OR = 0.78, 95% CI: 0.46–1.34, *P *=* *0.37). Overall, non‐LIS patients had a lower initiation rate for any targeted therapies as compared to LIS patients. Sensitivity analyses showed consistent findings (Table 4).

**Table 3:** Targeted therapy initiation rates among fee‐for‐service Medicare patients newly diagnosed with metastatic renal cell carcinoma, by low‐income subsidy status

	Observed initiation rate (No. Initiating/No. Patients)			Adjusted initiation rate		OR (95% CI)	*P*‐value^b^
	Non‐LIS	LIS	*P*‐value^a^	Non‐LIS (%)	LIS (%)		
Part D targeted therapies	291/1399	106/322	<0.001	20.7	33.9	0.49 (0.36–0.67)	<0.001
Part B targeted therapies	113/1399	35/322	0.110	8.2	10.2	0.78 (0.46–1.34)	0.370
Part D or Part B targeted therapies	373/1399	130/322	<0.001	26.7	40.4	0.52 (0.38–0.71)	<0.001

CI, confidence interval; LIS, low‐income subsidy; OR, odds ratio.

a *P*‐values for observed rates were based on chi‐square tests.

b *P*‐values for adjusted rates and odds ratios were based on logistic regressions adjusted for all covariates listed in Table 2.

**Table 4:** Sensitivity analyses, rates of targeted therapy initiation among patients newly diagnosed with metastatic renal cell carcinoma who were not receiving low‐income subsidies^a^

	N	Part D Initiation		Part B Initiation		Part D or B Initiation	
		OR (95% CI)	*P*‐value	OR (95% CI)	*P*‐value	OR (95% CI)	*P*‐value
Plan formulary fixed effect models	1721	0.45 (0.33–0.63)	<0.001	0.74 (0.40–1.37)	0.34	0.48 (0.35–0.67)	<0.001
Including patients diagnosed with mRCC during any Part D coverage phase	1954	0.50 (0.35–0.72)	<0.001	0.94 (0.55–1.59)	0.82	0.54 (0.38–0.77)	<0.001
Including patients with ≥2 metastatic claims, even if not 30 days apart	1953	0.57 (0.44–0.75)	<0.001	0.87 (0.52–1.46)	0.59	0.62 (0.47–0.82)	<0.001
Including patients with a stay in a skilled nursing facility	1805	0.48 (0.37–0.62)	<0.001	0.77 (0.47–1.24)	0.28	0.50 (0.38–0.66)	<0.001
Only controlling for statistically significant covariates	1721	0.55 (0.45–0.68)	<0.001	0.74 (0.48–1.16)	0.19	0.55 (0.45–0.68)	<0.001

CI, confidence interval; mRCC, metastatic renal cell carcinoma; OR, odds ratio.

a Reference group is patients who were receiving full low‐income subsidies.

**Figure 1:**
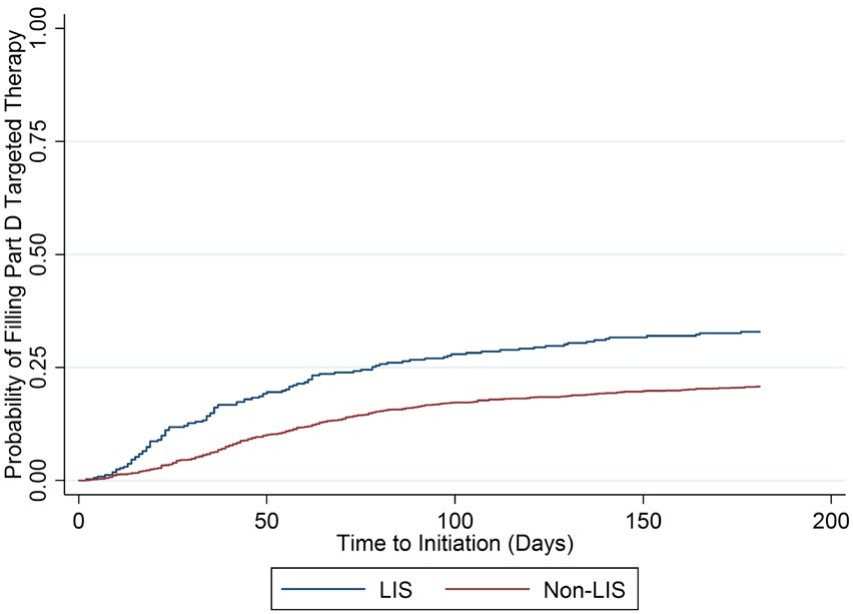
Kaplan–Meier curves for time to targeted therapy initiation in days, by low‐income subsidy status. Cox regression controlling for all covariates listed in Table 2 showed hazard ratio of 0.52 (95% CI: 0.38–0.71, *P *< 0.001). LIS, low‐income subsidy.

**Figure A2:**
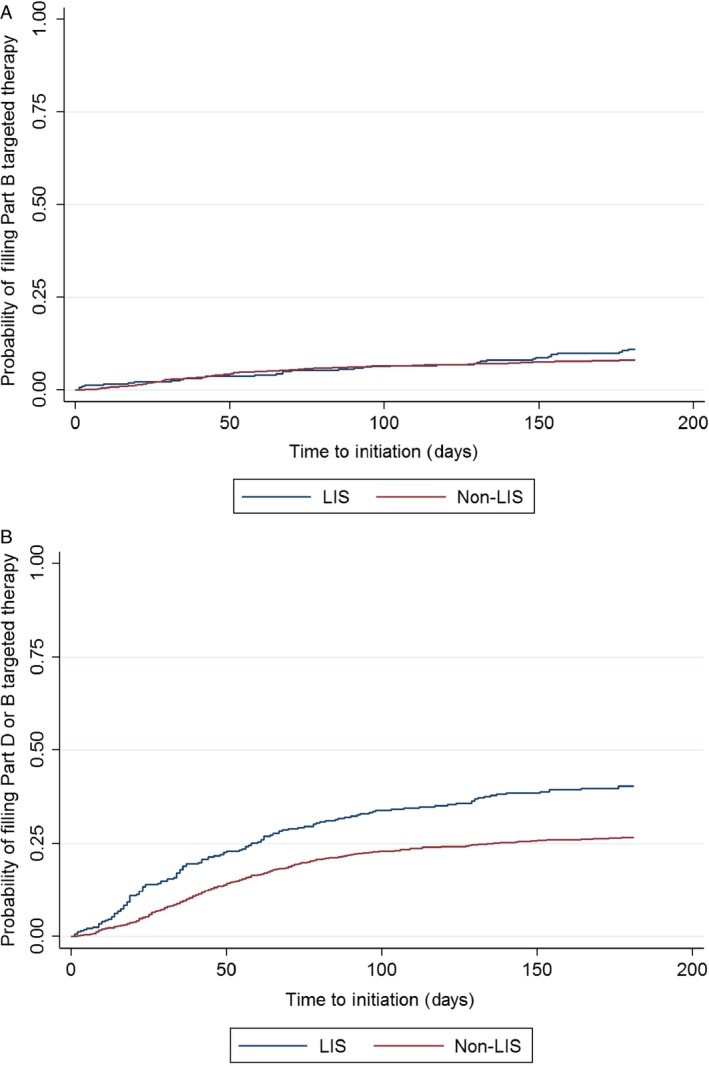
Kaplan–Meier curves for time to targeted therapy initiation, by low‐income subsidy status (A). Time to Part B targeted therapy initiation, in days. Cox regression controlling for all covariates listed in Table 2 showed hazard ratio of 0.80 (95% CI:0.49‐1.34, *P* = 0.408). (B) Time to Part D or B targeted therapy initiation, in days. Cox regression controlling for all covariates listed in Table 2 showed hazard ratio of 0.58 (95% CI:0.44‐0.76, *P* < 0.001). LIS, low‐income subsidy.

There was no significant difference in time to initiation for Part B targeted therapies between the non‐LIS and LIS groups, but it took longer for non‐LIS patients to access Part D targeted therapies and any targeted therapy (Fig. 1, Fig. A2(a‐b), and Table A1).

## Discussion

In the first 6 months after a new mRCC diagnosis, we found significantly lower initiation rates for Part D oral targeted therapies among elderly Medicare beneficiaries who were responsible for high out‐of‐pocket costs, as compared to their counterparts who faced minimal out‐of‐pocket costs due to receipt of low‐income subsidies (LIS). The association between high cost sharing and reduced rates of treatment initiation was apparent despite controlling for demographic and clinical characteristics that might influence treatment decisions and was confirmed via several sensitivity analyses. In addition, even though non‐LIS patients typically face lower out‐of‐pocket costs for infused targeted therapies available through their Part B medical benefit 12, we did not find higher initiation rates for Part B drugs among non‐LIS patients; non‐LIS patients had significantly lower rates of initiating *any* targeted therapies, as compared to LIS individuals. Furthermore, non‐LIS patients were slower to initiate therapy, as compared to their LIS peers. These results are consistent with our prior findings that Medicare patients newly diagnosed with chronic myeloid leukemia demonstrated both reduced and delayed initiation of life‐saving specialty drugs when responsible for high out‐of‐pocket costs under Part D 18.

It is worth noting that 6‐month targeted therapy initiation rates ranged from 27 to 40% in our newly diagnosed mRCC sample, even among those facing minimal cost sharing. It is unclear if these rates are lower than might be expected;^19^ although we attempted to capture patients who would be suitable candidates for targeted therapies, claims data do not include the complete range of clinical details that may figure into treatment decisions. For instance, we were unable to identify cases where treatment initiation was postponed deliberately in favor of observation, palliative radiation, or metastasectomy 14. In addition, post‐hoc analyses revealed that eight patients classified as non‐initiators were using either an off‐label targeted therapy (i.e., erlotinib) or other therapy (e.g., interleukin‐2). Nonetheless, we would not expect systematic differences in treatment decision‐making between the non‐LIS and LIS groups, so the discrepancy in initiation rates may still be associated with cost‐sharing differences.

Several other limitations should be noted. This was an observational, cross‐sectional analysis that documented associations but did not establish a causal relationship between high cost sharing and treatment initiation. We employed multivariable regression to control for sociodemographic, clinical, plan, and county‐level characteristics that could influence treatment decisions, yet unobserved confounding related to variables not available in claims data (e.g., patient preferences, additional clinical factors) could have contributed to the observed differences between groups. In addition, we chose the 100% CCW files because they permitted access to a larger sample of patients and linkage to Part D plan and formulary characteristics data, but our sample may have failed to capture patients with missing clinical codes for metastases in the claims. CCW files also do not include the tumor registry data available in Surveillance, Epidemiology and End Results (SEER)‐Medicare files. Therefore, we sought to identify patients with newly diagnosed metastatic disease but did not have access to stage at initial presentation. Patients initially treated for localized disease who later developed small metastases detected on imaging might have a more indolent course than patients who presented with de novo metastatic disease and thus might be less likely to require immediate pharmacological treatment. We do not have reason to believe that this would vary systematically between LIS and non‐LIS patients, however. In fact, post‐hoc analyses failed to detect systematic differences in nonpharmacological treatment between groups. Similar percentages of LIS and non‐LIS patients had a claim for inpatient surgery in the pre‐index (6.1% vs. 7.1%, *P *=* *0.51) and post‐index periods (35.9% vs. 32.3%, *P *=* *0.23), and similar percentages of patients in the LIS and non‐LIS groups received radiation therapy in the pre‐index (12.7% vs. 13.4%, *P *=* *0.76) and post‐index periods (34.9% vs. 30.1%, *P *=* *0.10).

As with all studies, the generalizability of our findings is directly related to our selection criteria. Requiring the new mRCC diagnosis to occur during Medicare Part D's initial coverage phase may have captured a healthier patient population (i.e., without substantial drug spending on other conditions), but a sensitivity analysis removing this restriction showed consistent results. We also restricted our analysis to patients with initial metastases in the liver, lung, or bone, and thus our results may not be representative of other metastatic sites 13. Finally, our study only included fee‐for‐service patients given CCW claims are available only for this sample, so our results may not generalize to Medicare Advantage beneficiaries or individuals receiving retiree drug coverage.

In addition, some patients receive assistance with prescription drug costs and this could have influenced our results in two ways. First, patients receiving copayment assistance through nonprofit foundations or other sources would have a Part D claim, but if they would not have been able to afford to initiate the medication without such assistance, our results would underestimate the true association between Part D cost sharing and treatment initiation. Second, if patients obtained medication outside of their Part D benefit (e.g., through a manufacturer program providing free medication), there would be no Part D claim and they would be erroneously classified as not initiating or delaying treatment 20. Although this could lead us to underestimate initiation rates overall, our results should still accurately reflect barriers to access and utilization under the Part D program.

Our study included a diverse group of patients covered by a wide range of Medicare plans, and we controlled for a wide variety of factors aside from cost that could influence treatment initiation. These claims‐based findings add to the conversation regarding financial barriers to treatment in oncology and complement valuable data and insights that have been gathered directly from patients. It is well‐established that cancer care is associated with substantial financial toxicity for many individuals and families 21, 22, and our findings suggest that even the threat of financial burden may be limiting treatment access. Although many factors influence treatment choice and initiation, the reduced utilization we observed among the high cost‐sharing group highlights the fact that despite the survival advantages offered by targeted therapies, not all patients may be able to access them promptly. Delays may have clinical significance, particularly for patients with symptomatic and/or rapidly progressing disease.

Our findings have both clinical and policy implications. In keeping with calls for providers to address financial toxicity 23, 24, our findings highlight the importance of proactive discussions about financial barriers when reviewing treatment options with patients. This is likely to be valuable even when patients do not raise financial concerns, since patients may not be aware of the out‐of‐pocket costs associated with treatment options. Furthermore, although our study examined treatment initiation, financial burden is likely to remain as a potential threat to subsequent adherence and may need to be a part of ongoing discussions between clinicians and patients 25.

At the systems level, streamlined processes for accessing copayment assistance are likely to be useful. One study found that more than one‐third of patients needed financial assistance before starting oral therapies 26, requiring multiple phone calls among patients, office staff, specialty pharmacies, and financial assistance programs and a median of 14 days from prescription to initiation for patients with mRCC 26. Reducing stress and burden associated with obtaining financial assistance is important.

The American Society of Clinical Oncology has called for policymakers to consider alternative benefit designs for life‐sustaining cancer treatments 1, and our findings suggest that longitudinal studies are needed to shed further light on the potential benefits of previously proposed strategies to address these issues, including value‐based insurance design approaches that reduce cost sharing for treatments that confer clinical advantages and implementation of annual and monthly maximum out‐of‐pocket spending limits under Medicare Part D 27, 28. As oncology treatment continues to move toward outpatient therapies, there is an increasing need to identify and reduce barriers to optimal outcomes and to examine how delays or interruptions in care impact clinical outcomes and overall health care costs.

## Conflict of Interest

Dr. Wong is now an employee at Janssen. At the time of the study, she was at Fox Chase Cancer Center and received funding from Pfizer. At the time of the study, Dr. Doshi reported serving as a consultant for Alkermes, Allergan, Ironwood Pharmaceuticals, Shire, and Vertex Pharmaceuticals; and had received research funding from AbbVie, Biogen, Humana, Janssen, PhRMA, Pfizer, Regeneron, and Sanofi. Dr. Doshi's spouse holds stock in Merck and Pfizer. Dr. Li, Mr. Jahnke, and Dr. Pettit have no conflicts to report.

## Appendix

**Table A1:** Cox regression on time to targeted therapy initiation among fee‐for‐service Medicare patients newly diagnosed with metastatic renal cell carcinoma

	Time to Part D targeted therapy		Time to Part B targeted therapy		Time to Part D or B targeted therapy	
	HR (95% CI)	*P*‐value	HR (95% CI)	*P*‐value	HR (95% CI)	*P*‐value
LIS status						
LIS	Reference		Reference		Reference	
Non‐LIS	0.52 (0.38–0.71)	<0.001	0.81 (0.49–1.34)	0.408	0.58 (0.44–0.76)	<0.001
Age category, years						
65‐69	Reference		Reference		Reference	
70–74	0.89 (0.68–1.16)	0.392	1.13 (0.74–1.74)	0.568	0.93 (0.73–1.17)	0.527
75–79	0.80 (0.60–1.07)	0.130	1.11 (0.71–1.74)	0.653	0.86 (0.67–1.10)	0.221
>80	0.70 (0.52–0.94)	0.020	0.45 (0.25–0.80)	0.007	0.61 (0.47–0.81)	<0.001
Sex						
Female	Reference		Reference		Reference	
Male	1.03 (0.84–1.26)	0.804	0.91 (0.65–1.28)	0.600	1.00 (0.83–1.20)	0.981
Race/ethnicity						
White	Reference		Reference		Reference	
Hispanic	1.17 (0.63–2.20)	0.617	1.09 (0.38–3.19)	0.868	1.22 (0.70–2.13)	0.490
Black	0.64 (0.40–1.01)	0.058	0.74 (0.36–1.55)	0.428	0.63 (0.42–0.96)	0.031
Other	1.18 (0.71–1.96)	0.526	0.72 (0.26–2.04)	0.542	1.02 (0.63–1.64)	0.933
Region						
West	Reference		Reference		Reference	
Midwest	0.86 (0.63–1.19)	0.368	0.72 (0.42–1.23)	0.226	0.83 (0.62–1.10)	0.200
Northeast	0.49 (0.33–0.73)	<0.001	0.95 (0.53–1.69)	0.850	0.57 (0.41–0.81)	0.001
South	1.02 (0.77–1.36)	0.892	0.94 (0.59–1.51)	0.797	1.00 (0.77–1.29)	0.986
Site of first metastasis^a^						
Liver	0.87 (0.53–1.43)	0.585	1.25 (0.61–2.57)	0.544	0.88 (0.57–1.37)	0.582
Lung	1.52 (0.97–2.37)	0.070	1.30 (0.65–2.60)	0.464	1.40 (0.93–2.09)	0.104
Bone	1.43 (0.92–2.22)	0.114	1.13 (0.56–2.25)	0.735	1.28 (0.86–1.91)	0.218
First metastases involved multiple sites						
No	Reference		Reference		Reference	
Yes	1.05 (0.79–1.41)	0.733	1.21 (0.77–1.90)	0.405	1.09 (0.85–1.41)	0.487
Charlson Comorbidity Index score	0.95 (0.88–1.03)	0.210	0.95 (0.83–1.08)	0.413	0.96 (0.89–1.03)	0.212
Part D drug benefit type^b^						
Enhanced alternative	Reference		Reference		Reference	
Basic alternative	0.92 (0.69–1.22)	0.554	1.07 (0.68–1.68)	0.773	0.93 (0.73–1.19)	0.568
Defined standard benefit	1.30 (0.67–2.50)	0.441	0.68 (0.15–3.06)	0.611	1.27 (0.69–2.33)	0.439
Other	0.92 (0.66–1.29)	0.632	1.24 (0.74–2.09)	0.414	0.98 (0.73–1.32)	0.890
Part D plan formulary characteristics						
Proportion of targeted therapies on market covered on the plan formulary	3.45 (0.62–19.31)	0.159	1.21 (0.09–16.86)	0.888	2.06 (0.46–9.15)	0.344
Proportion of covered targeted therapies requiring prior authorization	1.10 (0.63–1.92)	0.748	1.17 (0.43–3.17)	0.762	1.06 (0.65–1.76)	0.805
Proportion of covered targeted therapies subject to quantity limits	1.04 (0.75–1.45)	0.812	1.55 (0.91–2.65)	0.106	1.15 (0.86–1.54)	0.351
Zip code–level variables						
Median household income	0.97 (0.91–1.04)	0.381	0.93 (0.84–1.04)	0.218	0.97 (0.91–1.02)	0.263
Percentage of those aged ≥25 with at least a high school degree	1.01 (0.99–1.02)	0.236	1.00 (0.98–1.03)	0.755	1.01 (0.99–1.02)	0.280
Year of mRCC diagnosis						
2011	Reference		Reference		Reference	
2012	1.01 (0.79–1.29)	0.952	0.88 (0.59–1.31)	0.515	0.93 (0.75–1.16)	0.517
2013	1.35 (1.03–1.77)	0.032	1.21 (0.78–1.89)	0.395	1.26 (0.99–1.60)	0.062

CI, confidence interval; HR, hazard ratio; LIS, low–income subsidy; mRCC, metastatic renal cell carcinoma.

a Categories are not mutually exclusive; patients were assigned to multiple categories if the first date of reported metastatic diagnosis in medical claims included multiple sites.

b Defined standard benefit has an annual deductible, 25% coinsurance in the initial coverage phase, and 45% cost sharing during the coverage gap; basic alternative may have reduced or $0 deductible, can use tiered copayments or coinsurance, and must be actuarially equivalent to the defined standard benefit; enhanced alternative exceeds the value of standard coverage and may include reduction/elimination of the initial deductible, an increase in the initial coverage limit, or a reduction of cost sharing in the coverage gap.
